# 

**Published:** 1914-10-17

**Authors:** 


### Buildings Contemplated.

Langstone.—The Portsmouth Town Council have decided to build a tuberculosis hospital at Langstone for forty patients, at a cost of £6,773, the Treasury contributing £4,064.

Little Cairnie.—The Arbroath Public Health Committee have considered the plans of the smallpox hospital to be built at Little Cairnie; they have agreed to remit the amended plans to the Local Government Board, and on receiving the approval of that body building operations will be commenced.

Nottingham.—The Corporation Estates Committee propose to lease land adjoining the Arboretum refreshment-rooms to the Trustees of the Hospital for Women, Castle Gate, for a new hospital.

Wallsend.—In connection with the proposed extensions at the hospital, the Town Council have decided that they shall consist of a new wing for scarlet-fever patients and extensions to the administrative block.

Waterford Ghyll.—The Skipton Rural District Council have decided to borrow £1,000 for the purchase of a site near Waterford Ghyll for a smallpox hospital.

### Medical Appointments.

The Mental Hospitals Committee of the London County Council report that Mr. T. D. O'Flynn, fourth assistant medical officer at Cane Hill Mental Hospital, has been appointed to be third assistant medical officer at Hanwell Mental Hospital, to succeed Dr. Petrie; and Dr. R. S. Miller, fifth assistant medical officer at Colney Hatch Mental Hospital, has been promoted to be fourth assistant medical officer.

RATES OF SUBSCRIPTION: Twelve months, 6/6; Six months, 4/-; Three months, 2/-, post free to any address in the United Kingdom. Abroad, Twelve months, 8/8; Six months, 5/-; Three months, 2/6, post free.—Address The Subscription Dept., "THE HOSPITAL," The Hospital Building, 28/29 Southampton Street, Strand, London W.C.

